# Late assessment of quality of life in patients with rectal carcinoma: comparison between sphincter preservation and definitive colostomy

**DOI:** 10.1007/s00384-018-3044-4

**Published:** 2018-04-19

**Authors:** Mariane Messias Reis Lima Silva, Samuel Aguiar Junior, Juliana de Aguiar Pastore, Érica Maria Monteiro Santos, Fábio de Oliveira Ferreira, Ranyell Matheus S. B. Spencer, Vinicius F. Calsavara, Wilson Toshihiko Nakagawa, Ademar Lopes

**Affiliations:** Department of Colorectal Tumors, AC Camargo Cancer Center, Professor Antonio Prudente, 211, Sao Paulo, SP 01509-900 Brazil

**Keywords:** Rectal cancer, Quality of life, Low anterior resection, Abdominoperineal resection, Colostomy

## Abstract

**Purpose:**

Patients with cancer of the lower and middle rectum who are candidates for curative surgery often have negative opinions on definitive colostomy. The purpose of this study is to compare the quality of life (QoL) of patients who undergo standard treatment for rectal cancer with sphincter preservation or definitive colostomy.

**Methods:**

A total of 125 patients with adenocarcinoma of the lower or middle rectum who underwent radical surgery with curative intent with a follow-up ≥ 1 year were recruited: 83 patients (group 1) were subjected to low anterior resection and low colorectal or coloanal anastomosis—thus preserving their sphincter—and 42 (group 2) were treated with abdominoperineal resection, followed by terminal definitive colostomy. QoL was assessed with the EORTC QLQ-C30 and QLQ-CR29 questionnaires.

**Results:**

Health and global quality of life were similar between groups; however, patients who underwent definitive colostomy had higher scores on the emotional (*p* value = 0.016) and cognitive function scales (*p* value = 0.017). Patients with sphincter preservation presented with more symptoms that were related to stool frequency (*p* value < 0.001), intestinal constipation (*p* value = 0.005), fecal incontinence (*p* value = 0.001), buttock pain (*p* value = 0.023), and nausea and vomiting (*p* value = 0.036), whereas patients with permanent colostomy had higher scores for dysuria (*p* value = 0.033).

**Conclusion:**

Although global QoL scores did not differ between groups, patients who underwent definitive colostomy had significantly better functional and symptom scale scores, reflecting greater function with fewer symptoms.

**Electronic supplementary material:**

The online version of this article (10.1007/s00384-018-3044-4) contains supplementary material, which is available to authorized users.

## Introduction

The incidence of colorectal cancer (CRC) has risen gradually in the past several decades. CRC ranks third highest in incidence among noncommunicable diseases and is the fourth leading cause of mortality worldwide [1, 2]. The number of living people diagnosed with cancer, including the cancer survivors, also increased, which reflects an increased number of people who were cured or who live longer with the disease [3], necessitating studies that analyze issues that are related to the quality of life (QoL) of survivors.

Neoadjuvant chemoradiation of patients with locally advanced lower and middle rectal tumors (cT3/T4 or N+) reduce local recurrence rates and contribute to sphincter preservation (SP) [4]. Nevertheless, there is interest in developing more conservative therapies that focus on selecting patients for treatments that are based exclusively on chemoradiation [5, 6]. Despite our knowledge of the outcomes of conservative treatment protocols, surgery with total mesorectal excision (TME) and adequate margins continues to be the most important component of a curative treatment strategy.

Thus, significant technical effort has been dedicated toward sphincter preservation, as evidenced by advances in laparoscopic colorectal surgery and robotics [7]. Consequently, low anterior resection of the rectum (LAR) with coloanal anastomosis and anatomical sphincter preservation has become a common practice to avoid abdominoperineal resection (APR) and definitive colostomy (DC). Although the sphincter is preserved anatomically, its functional preservation has been insufficiently assessed. Despite the technological evolution, APR that is followed by definitive colostomy continues to be the preferred alternative for patients with distal rectal lesions with anal sphincter invasion or expectancy of severe sphincter malfunction after LAR and coloanal anastomosis [8]. In this regard, many studies have suggested that patients with a stoma and those with anatomical sphincter preservation experience significant changes in the physical, social, and physiological aspects of their QoL [9–12].

This report is the first study in Latin America at a large cancer center with a representative patient sample to examine QoL in patients with a definitive stoma compared with patients with distal anastomosis after LAR. Our main goal was to compare the QoL between rectal adenocarcinoma patients who are treated with low anterior resection with sphincter preservation and those who undergo abdominoperineal resection with definitive colostomy using statistical methods.

## Methods

This observational, cross-sectional, and comparative quantitative study was conducted from April 2012 to June 2013. Patients who were regularly scheduled for follow-up during this period were invited to participate. The inclusion criteria were the following: patients aged over 18 years, follow-up after previous treatment for rectal carcinoma, subjected to radical surgery with TME by the Colorectal Cancer Service of AC Camargo Cancer Center with or without sphincter preservation, and at least 12 months after APR or after closure of a diverting stoma. The exclusion criteria were the following: patients with distinctive stomas from definitive terminal colostomy and histology that differed from adenocarcinoma and intraperitoneal rectal tumors.

During the study period, 137 patients were identified, and after being pared due to exclusion criteria (*n* = 4), refusal to participate (*n* = 1), and loss to follow-up (*n* = 7), the study population ultimately comprised 125 patients. The median follow-up was 3.84 years. The patients were divided into two groups: (1) the sphincter preservation group (SPG) contained 83 patients (66%) who were subjected to LAR and low colorectal or coloanal anastomosis and anatomic sphincter preservation; and (2) the definitive colostomy group (DCG) was composed of 42 patients (34%) who underwent APR and terminal definitive colostomy.

QoL assessments were performed on completion of the validated European Organization for Research and Treatment of Cancer (EORTC) questionnaires: QLQ-C30 and QLQ-CR29. The Quality of life Questionnaire-Core Questionnaire (C30) is a generic instrument and consists of 30 questions on physical, life role, cognitive, emotional, and social functioning; it also includes three symptom scales (fatigue, nausea and vomiting, pain); six individual questions on dyspnea, loss of appetite, insomnia, constipation, diarrhea, and financial difficulties; and two questions on general QoL and health. QLQ-CR29 is a module that specifically evaluates patients with colorectal cancer comprises 29 questions on colorectal disease-specific symptoms and function. The scores range from 0 to 100, wherein higher scores on the functional scale reflect better health or QoL; high scores on the symptom scale indicate a significant level of problems [13–15]. A social, demographic, and clinical form was also administered.

Initially, the clinical and demographic variables and quality of life were analyzed using descriptive statistics. To determine whether there was a relationship between two categories, chi-square test for independence was applied. To compare continuous variables in relation to the two groups (DC and SP), we used *t* test or Mann-Whitney *U* test, where applicable. To identify risk factors that were associated with QoL, we fitted generalized linear regression models with gamma and inverse Gaussian-distributed-dependent variables. The significance level was 5%. All statistical analyses were performed in R, version 3.2.

The study was approved by the institutional ethics committee (IRB) by the ID 01610/11. The study is also registered at the governmental Brazilian registry of scientific projects (Plataforma Brasil) by the ID 0089.0.022.000-11.

## Results

The social, demographic, and clinical characteristics of the SPG and DCG are compared in Table 1. The DCG had a higher proportion of lower rectal tumors (78.6 vs. 41.0%; *p* value < 0.001) and received pelvic radiotherapy more frequently (92.7 vs. 75.3%; *p* value = 0.015) than SPG patients. There was no significant difference between groups with regard to age, gender, race, education, participation in religious groups, active employment, marital status, or social status.

**Table 1:** Social, demographic, and clinical characteristics between SPG (sphincter preservation group) and DCG (definitive colostomy group)

Variable	Category	SPG (*n =* 83)	DCG (*n =* 42)	*p* value
Age (in years)	Mean (SD)	60.12 (11.48)	64.10 (12.54)	0.079
Gender	Male	40 (48.2%)	23 (54.8%)	0.488
	Female	43 (51.8%)	19 (45.2%)	
Race	White	65 (78.3%)	30 (71.4%)	0.395
	Non-white	18 (21.7%)	12 (28.6%)	
Education level	High school or less	52 (62.7%)	23 (54.8%)	0.395
	Graduated or higher	31 (37.3%)	19 (45.2%)	
Religious group	No	27 (40.3%)	12 (34.3%)	0.553
	Yes	40 (59.7%)	23 (65.7%)	
Active employment	No	37 (44.6%)	24 (57.1%)	0.184
	Yes	46 (55.4%)	18 (42.9%)	
Marital status	Stable union/married	61 (73.5%)	28 (66.7%)	0.426
	Divorced/widowed/single	22 (26.5%)	14 (33.3%)	
Social status	L, ML, and M	45 (73.8%)	26 (78.8%)	0.589
	MH and H	16 (26.2%)	7 (21.2%)	
Rectum tumor localization	Lower	33 (39.8%)	34 (81.0%)	< 0.001
	Middle	50 (60.2%)	8 (19.0%)	
Pelvic radiotherapy	No	20 (25.0%)	3 (7.1%)	0.017
	Yes	60 (75.0%)	39 (92.9%)	

*L*, low; *ML*, medium-low; *M*, middle; *MH*, medium-high; *H*, high

### Assessment of quality of life

Our analysis of the EORTC QLQ-C30 questionnaires showed no differences between groups in global QoL (*p*-value = 0.678). There were significant differences in functional scale scores, with higher values observed for patients with definitive colostomy in the emotional (*p* value = 0.016) and cognitive domains (*p* value = 0.017). No significant differences were seen in the physical, functional role, and social scales. On the symptom scales, significant differences were noted between groups (SP, CD) for nausea and vomiting (*p* value = 0.036) and constipation (*p* value = 0.005), whereas fatigue, pain, dyspnea, insomnia, loss of appetite, diarrhea, and financial difficulty scores were similar (Table 2).

**Table 2:** Quality of life analysis: comparison of median scores between SPG and DCG for domains assessed by the EORTC QLQ-C30

EORTC QLQ C-30	SPG (*n* = 83) median (min.–max.)	DCG (*n* = 42) median (min.–max.)	*p* value
Physical functioning	86.7 (20.0–100.0)	73.3 (13.3–100.0)	0.133
Role functioning	83.3 (0.0–100.0)	83.3 (16.7–100.0)	0.314
Emotional functioning	66.7 (8.3–100.0)	75.0 (0.0–100.0)	0.016
Cognitive functioning	83.3 (0.0–100.0)	91.7 (0.0–100.0)	0.017
Social functioning	100.0 (0.0–100.0)	91.7 (0.0–100.0)	0.497
Fatigue	22.2 (0.0–100.0)	16.7 (0.0–66.7)	0.680
Nausea and vomiting	0.0 (0.0–100.0)	0.0 (0.0–33.3)	0.036
Pain	16.7 (0.0–100.0)	0.0 (0.0–100.0)	0.535
Dyspnea	0.0 (0.0–100.0)	0.0 (0.0–66.7)	0.682
Insomnia	33.3 (0.0–100.0)	33.3 (0.0–100.0)	0.379
Appetite loss	0.0 (0.0–100.0)	0.0 (0.0–100.0)	0.208
Constipation	0.0 (0.0–100.0)	0.0 (0.0–100.0)	0.005
Diarrhea	0.0 (0.0–100.0)	0.0 (0.0–66.7)	0.198
Financial difficulties	0.0 (0.0–100.0)	0.0 (0.0–100.0)	0.585
Global health and QoL	75.0 (0.0–100.0)	75.0 (0.0–100.0)	0.678

Scores range from 0 to 100. On the functional scales, higher scores indicate a better QoL; on the symptom scales, lower scores reflect a better QoL

Based on QLQ-CR29 scores, there were no significant differences between groups on the functional scales—body image, anxiety, weight dissatisfaction, and sexual interest. However, patients with sphincter preservation had higher scores—i.e., experiencing worse symptoms for buttock pain (*p* value = 0.023), fecal incontinence (*p* value = 0.001), and bowel movement frequency (*p* value = 0.001) than DCG subjects, who in turn had higher scores for dysuria (*p* value = 0.033) (Table 3).

**Table 3:** Quality of life analysis: comparison of median scores between SPG and DCG on the EORTC QLQ-CR29

EORTC QLQ-CR29 Functional scales	SPG (*n* = 83) median (min.–max.)	DCG (*n* = 42) median (min.–max.)	*p* value
Body image	88.9 (0.0–100.0)	86.1 (0.0–100.0)	0.987
Anxiety	66.7 (0.0–100.0)	33.3 (0.0–100.0)	0.279
Weight	66.7 (0.0–100.0)	66.7 (0.0–100.0)	0.847
Sexual interest- men	33.3 (0.0–100.0)	33.3 (0.0–100.0)	0.686
Sexual interest- women	66.7 (33.3–100.0)	66.7 (0.0–100.0)	0.458
Urinary frequency	33.3 (0.0–100.0)	41.7 (0.0–83.3)	0.362
Urinary incontinence	0.0 (0.0–100.0)	0.0 (0.0–100.0)	0.198
Dysuria	0.0 (0.0–66.7)	0.0 (0.0–100.0)	0.033
Abdominal pain	0.0 (0.0–100.0)	0.0 (0.0–66.7)	0.096
Buttock pain	0.0 (0.0–100.0)	0.0 (0.0–100.0)	0.023
Abdominal pain	0.0 (0.0–100.0)	0.0 (0.0–100.0)	0.238
Dry mouth	0.0 (0.0–100.0)	0.0 (0.0–100.0)	0.825
Hair loss	0.0 (0.0–100.0)	0.0 (0.0–33.3)	0.502
Taste	0.0 (0.0–100.0)	0.0 (0.0–100.0)	0.141
Flatulence	33.3 (0.0–100.0)	33.3 (0.0–100.0)	0.998
Fecal incontinence	33.3 (0.0–100.0)	0.0 (0.0–100.0)	0.001
Sore skin	0.0 (0.0–100.0)	0.0 (0.0–100.0)	0.345
Stool frequency	33.3 (0.0–100.0)	0.0 (0.0–66.7)	0.001
Embarrassment	0.0 (0.0–100.0)	0.0 (0.0–100.0)	0.584
Impotence	66.7 (0.0–100.0)	100.0 (0.0–100.0)	0.101
Dyspareunia	0.0 (0.0–100.0)	0.0 (0.0–100.0)	0.705

Scores range from 0 to 100. On functional scales, higher scores indicate a better QoL; on symptom/problem scales, lower scores reflect a better QoL

Independent variables—age, gender, social status, education level, active employment, pelvic radiotherapy, colostomy, and sphincter preservation—were fitted to generalize linear models of the data in relation to quality of life. However, no independent variable was sufficiently significant to explain QoL scores.

## Discussion

The main findings of this study were the similar global QoL scores between groups (CD, SP) for the QLQ-C30 questionnaire and the greater frequency of anorectal problems that were reported by patients with sphincter preservation on the QLQ-CR29 instrument. Other studies have corroborated this equivalence in global health status after late surgical follow-up [16–19].

VARPE et al. [20] compared the QoL of patients who underwent APR, LAR, and intersphincter resection and also failed to note any significant differences in global QoL scores between groups. In a meta-analysis of 11 studies on QoL in 1433 patients—33% with DC and 67% with SP—with an average follow-up of 15 months after surgery, CORNISH et al. [21] did not observe any significant differences in global QoL scores between groups. Thus, FUCINI et al. [22] reported distinct results using the EORTC QLQ-C30 and QLQ-CR38 instruments in 30 patients with DC and 32 subjects with SP, the latter of whom had better global QoL scores, nevertheless recurrent, postsurgery, and comorbid cases were excluded, and only patients were assessed after 5 years of follow-up; however, patients who survived for less than 5 years were excluded.

Notably, in our study, patients with definitive colostomy had better results with regard to emotional and cognitive functions and fewer symptoms for nausea and vomiting, constipation, fecal incontinence, bowel movement frequency, and buttock pain. These results are consistent with other studies that reported higher scores for emotional and cognitive functions in DC patients and more gastrointestinal problems after LAR [10, 12, 21]. In contrast, Monastyrska et al. [23] recorded better emotional and cognitive functional scale scores for SP subjects in a recent series with a follow-up of 6 months after surgery.

Emotional function is related to feelings, such as nervousness, concerns, irritability, and depression. The worsening of scores on this scale in patients with SP might be related to their symptoms of incontinence and constipation, increased frequency of bowel movement, and sensation of incomplete bowel movement, which can create insecurity and unease. Other studies have shown that after reversal of the stoma in patients with SP, the resulting alterations in their gastrointestinal systems significantly impacted their emotional health [10, 12, 24].

In Latin America, most studies on the influence of definitive colostomy have been qualitative, with small numbers of patients. The inclusion criteria of these studies included newly operated patients who were in the stages of adapting to the stoma and thus had such symptoms as peristomal dermatitis and psychological problems over accepting the use of a colostomy bag. Support by the family and health team is essential for the self-care and independence of these patients and their improvements in self-esteem [25–28]. In our study, patients had adapted to definitive colostomy, because they had 1 year or more to prepare for the stoma; thus, problems with stoma care, altered body image, and skin issues were less extensive.

Scores for nausea and vomiting were higher for patients with SP compared with DC subjects. Notably, 10 patients were being treated with chemotherapy during the study, 4 of whom reported experiencing nausea and vomiting symptoms in the SPG. However, other unknown causes could have interfered with these changes.

On the QLQ-CR29 instrument, patients with sphincter preservation had higher scores for symptoms of fecal incontinence, bowel movement frequency, and buttock pain and constipation, as reported by other groups [16, 17, 20, 29].

Fecal incontinence can be explained in part by the denervation that is caused by surgical intervention, particularly in the treatment of lower lesions, with damage to anal rectal function—some of it due radiotherapy whenever it is performed [30, 31]. According to Mulsow et al. [19], intersphincter resection yields the worst functional results, wherein the internal sphincter is cut. In a systematic review of 14 studies on intersphincter resection, Akagi et al. [8] found high rates of symptoms, such as bowel movement urgency, stool fragmentation, and fecal incontinence. Thus, patients with intersphincteric LAR are especially susceptible to developing “low anterior resection syndrome,” which incorporates unpleasant symptoms, such as frequent bowel movement, urgency, incontinence, constipation, and feelings of incomplete emptying [20, 30, 32–35].

Although patients with DC fail to control their bowel movement, it remains in the collection pouch when they become well adapted. In contrast, patients with LAR can develop bowel incontinence, causing leakages that can soil clothing and create unpleasant odors, causing embarrassment and discomfort and thus worsening emotional function.

Another factor that can interfere with gastrointestinal function is neoadjuvant treatment and adjuvant treatment with radiotherapy and chemotherapy, the use of which is associated with an increase in bowel movement frequency, higher rates of diarrhea^,^ urges and fecal incontinence, and reductions in quality of life and social functioning [32, 36–38]. In our study, 85.6% of patients received radiotherapy, of whom 45.8% reported fecal incontinence, 54.2% noted increased bowel movement frequency, 40.1% reported constipation, and 15% claimed to have dysuria.

In our series, the DCG had higher scores for dysuria. Notably, all patients who had dysuria received radiotherapy during the treatment, and the number of patients who were administered pelvic radiotherapy was significantly higher in the DCG. Other studies have also shown that patients who undergo neoadjuvant radiotherapy have higher scores on urinary problems versus those who do not and those who receive adjuvant radiotherapy [30, 31, 36, 37].

With regard to the items that are related to scales of sexual function, the low rate of responses, especially among women, did not allow us to make an adequate comparison between groups. Although the mean age of the women in this study was 56.46 years old, 1/3 of them were 60 years or older, so the low response rate can be explained due to an absence or decrease in the sexual life of the women with more advanced ages. Another factor that may have interfered with this lack of answers would be shame, since Brazil is a developing country, with typical cultural and religious influences, and the population is still uncomfortable to speak or expose itself with regard to sexuality issues. Other studies have also reported the absence of responses for questions that are related to sexuality [17, 20, 24, 31, 33, 36].

In conclusion, we did not observe any significant difference in global QoL scores between patients with lower and middle rectal adenocarcinoma who underwent surgical treatment with sphincter preservation or definitive colostomy. The DCG scored significantly better on several aspects, including the functional scales and certain items on the symptom scales, most frequently reporting fewer complaints that were related to fecal incontinence, frequency of bowel movement, intestinal constipation, and pain in the buttock area.

The main limitation of our study is the retrospective nature of the cohort. We have collected QOL data prospectively, but during a late post-treatment moment. We do not have QOL data before treatment, and all treatment-related data (chemoradiation toxicity, post-operative complications) are retrospective, although well collected and easily available in our institutional databank. Ideally, we should had done a prospective cohort study, but this would require a longer time to perform the research. A cross-sectional design as we have performed allowed us to achieve significant results quickly, from a pre-existing cohort. Our current results, although these limitations, encourage us strongly to design a prospective and more robust study. Other limitation is the relatively small sample size. In a prospective design, we certainly should consider a multi-institution study, for recruiting a larger number of patients.

Our results contribute to a better counseling of patients with low and middle rectal cancer who are candidates for curative surgical treatment with regard to the late postsurgical outcomes and QoL, dispelling negative ideas about definitive colostomy and demonstrating that DC is not so bad as seen. More studies should be performed to develop better treatment techniques for patients with low anterior resection syndrome and improved counseling and treatment protocols, to enhance their QoL.

## Electronic supplementary material

ESM 1 (XLSX 34 kb)
